# Evaluation of Neutralizing Capacity of Tixagevimab plus Cilgavimab (AZD7442) against Different SARS-CoV-2 Variants: A Case Report Study with Comparison to a Vaccinated Population

**DOI:** 10.1155/2024/9163490

**Published:** 2024-08-31

**Authors:** Constant Gillot, Jean-Louis Bayart, Vincent Maloteau, Jean-Michel Dogné, Jonathan Douxfils, Julien Favresse

**Affiliations:** ^1^ Clinical Pharmacology and Toxicology Research Unit Namur Research Institute for Life Sciences University of Namur, Namur 5000, Belgium; ^2^ Department of Laboratory Medicine Clinique St-Pierre, Ottignies, Belgium; ^3^ Department of Pharmacy University of Namur, Namur 5000, Belgium; ^4^ Qualiblood s.a. Research and Development Department, Namur, Belgium; ^5^ Department of Laboratory Medicine Clinique St-Luc, Bouge, Belgium

## Abstract

AZD7442 (150 mg of tixagevimab plus 150 mg of cilgavimab) has been approved for the preexposure prophylaxis of COVID-19 and for the treatment of adults and adolescents with COVID-19 who do not require supplemental oxygen and who are at increased risk of severe COVID-19. Thus, the aim of the present study is to evaluate the neutralizing capacity of tixagevimab and cilgavimab across different SARS-CoV-2 variants in two patients who received AZD7442 for immunoprophylaxis. A cohort of subjects (*n* = 45) who had received the BNT162b2 mRNA COVID-19 vaccine has been included to compare these two preventive strategies. Neutralizing antibody (NAb) titers against several variants were assessed against the wild-type, alpha, beta, gamma, delta, omicron BA.5, and XBB.1.5 variants. Binding antibodies have also been measured. NAbs *T*_1/2_ for AZD7442 was 8.1 days (95% CI: 5.1–19.5 days) and was 11.8 days (95% CI: 7.9–23.7 days) for the primo-vaccination cohort. The time to reach neutralization negativity was 108.3 days (95% CI: 66.9–130.7) for AZD7442 compared to 95.4 days (95% CI: 31.0–119.7 days) for the primo-vaccination cohort. The time to reach NAbs' negativity differs between variants with the maximum value obtained for alpha (i.e., 101.1 days (95% CI: 30.0–135.4 days)) and the minimum obtained for beta (i.e., 61.2 days (95% CI: 37.8–77.1 days)). Our results reinforce the need of reviewing the use of AZD7442 in relation to variants of concern and potentially adapting its administration schedule. AZD7442 could be indicated for short-term prophylaxis in frail patients who may be acutely exposed to SARS-CoV-2.

## 1. Introduction

Vaccination against severe acute respiratory syndrome coronavirus 2 (SARS-CoV-2) has reduced the burden of coronavirus disease 2019 (COVID-19) [1]. However, some patients, including immunocompromised persons such as recent transplant patients or patients suffering from lymphoid hemopathy, remain at risk for severe COVID-19 despite having been vaccinated with regular booster doses [2, 3]. There are several alternatives for these individuals such as the use of convalescent COVID-19 patient plasma (CCP) or the use of monoclonal antibodies (mAbs) against SARS-CoV-2. CCPs collected from recovered COVID-19 patients are supposed to be rich in neutralizing antibodies (NAbs) [4, 5]. CCP will almost always be the first antibody therapy available to treat an outbreak with a new SARS-CoV-2 variant [6]. They are injected just after the development of the symptoms and aim at treating the disease [4]. CCPs need to be analyzed to determine their neutralizing capacity before use, although the determination of their neutralizing capacity is not always related to the different variants of interest (VOIs). Focosi et al. reported that CCPs with NAb titer >160 are effective [7]. However, there are several limitations to the use of CCPs, such as injection timing, injection volume, which can be very large (up to 2,400 mL), and the phenomenon of antibody-dependent enhancement (ADE) [8]. Therefore, there is a need to have access to therapies which are at least as efficient as CCPs to protect individuals but without the need to continuously feed biobanks with plasma from donors, which is clearly also dependent on patient's recruitment. Monoclonal antibodies, which protect against disease irrespective of immune system status and provide rapid protection, are potential options for COVID-19 immunoprophylaxis [9, 10].

AZD7442, an association of 150 mg of tixagevimab and 150 mg of cilgavimab (Evusheld®, AstraZeneca, Södertälje, Sweden), has been approved for the preexposure prophylaxis of COVID-19 and for the treatment of adults and adolescents with COVID-19 who do not require supplemental oxygen and who are at increased risk of progressing to severe COVID-19 [11]. These mAbs are modified at their Fc regions with the aim to increase their half-life time (*T*_1/2_) [12]. In the PROVENT study, symptomatic COVID-19 occurred in 8 of 3,441 participants (0.2%) in the AZD7442 group and in 17 of 1,731 participants (1.0%) in the placebo group (relative risk reduction, 76.7%; 95% confidence interval (CI), 46.0 to 90.0; *p* < 0.001) supporting the claim that a single dose of AZD7442 showed efficacy for the prevention of COVID-19, without evident safety concerns. Based on these clinical data, the duration of protection following administration of a single AZD7442 dose is estimated to be at least 6 months [13].

Importantly, the clinical trial program with AZD7442 was conducted when alpha (B.1.1.7), beta (B.1.351), gamma (P.1), and delta (B.1.617.2, +K417N, and AY.1/AY.2) variants were predominant. According to the data provided by AstraZeneca, the combination of tixagevimab and cilgavimab retained full to nearly full neutralization activity against pseudovirus and/or live virus SARS-CoV-2 variant strains harboring all spike substitutions identified in alpha (B.1.1.7), beta (B.1.351), gamma (P.1), delta (B.1.617.2, +K417N, and AY.1/AY.2), and omicron (BA.2) [14–16]. Pseudotyped virus-like particles expressing spike protein and authentic SARS-CoV-2 omicron BA.1 variant (B.1.1.529) and omicron BA.1.1 (B.1.1.529, +R346K) showed, on the other hand, reduced susceptibility to the combination of tixagevimab plus cilgavimab [14–16]. Thus, the efficacy of tixagevimab and cilgavimab against some circulating SARS-CoV-2 variants with decreased *in vitro* susceptibility is still uncertain. Due to this observed decrease in neutralization activity against the omicron subvariants such as BA.4, BA.5, XBB 1.5, and recently XBB 1.16, the duration of the protective effect of AZD7442 for these subvariants is currently unknown.

As of today, only the omicron variant XBB 1.5 is considered as VOIs [17]. The emergence of these variants raised concerns regarding the duration of vaccine efficacy as assessed by comparing the residual neutralizing capacity of the immune response generated with vaccines encoding for the ancestral spike protein [18–25]. This observation has led the marketing authorization holders to adapt their vaccines to these VOIs. Such strategy could also apply to mAbs if there is evidence that their neutralizing capacity is significantly reduced with the current VOIs. Thus, the aim of the present study is to evaluate the neutralizing capacity of tixagevimab and cilgavimab across different SARS-CoV-2 variants.

## 2. Study Design and Population

Two patients received AZD7442 for COVID-19 immunoprophylaxis. One subject (female, 28 years) was seropositive to SARS-CoV-2 before the administration of AZD7442, i.e., antinucleocapsid antibody level over the positive threshold, and the other subject (female, 32 years) was seronegative, i.e., anti-nucleocapsid antibody level below the positive threshold [26]. Blood samples were obtained at different times after injection of AZD7442, i.e., 0, 7, 14, 28, 56, and 90 days following injection. No breakthrough infection occurred during this period in these AZD7442 patients.

To compare the immunity acquired following AZD7442 administration to another prophylactic strategy, a cohort from the CRO-VAX study was used. The CRO-VAX-HCP study is a Belgian multicenter, prospective, and interventional study that was designed to assess the humoral response in a population of healthcare workers (HCWs) from 18 to 65 years of age having received two doses of the BNT162b2 mRNA COVID-19 vaccine (Comirnaty, Pfizer-BioNTech) [27]. The study was approved by a central ethical committee (CHU UCL Namur, Yvoir, Belgium; approval number: 2020‐006149‐21). A total of 231 participants were initially enrolled. Participants provided written informed consent to take part in the study [28]. Only subjects without breakthrough infection were included. Only samples (*n* = 45) collected after 0, 7, 14, 28, 56, and 90 days after vaccination were included in this study to match the sampling scheme of AZD7442 participants [18, 29].

### 2.1. Analytical Procedures

#### 2.1.1. Neutralizing Antibodies

Neutralizing antibodies were analyzed using a pseudovirus neutralization test (pVNT). Pseudoviruses were from E-enzyme (Gaithersburg, MD, USA). SARS-CoV-2 pseudoviral particles are replication-deficient Maloney murine leukemia virus (MLV or MuLV) pseudotyped with the SARS-CoV-2 spike protein carrying a genotype depending on the variant used. They also contain the open reading frame (ORF) for firefly luciferase as a reporter. Briefly, HEK293T hACE2 cells (catalog *n*°: hkb-hace2, InvivoGen, CA, USA) were seeded at a density of 8,500 cells/well in a white 384-well cell culture plate. The sera used were heat-inactivated in a water bath at 54°C for 30 min and then serially diluted in a culture medium, Dulbecco's modified Eagle's medium (DMEM, catalog *n*°: L0102-500, VWR, PA, USA), supplemented with 10% of fetal bovine serum (FBS, catalog *n*°: S181B-100, VWR, PA, USA). Thereafter, samples are mixed in a 1 : 4 ratio with pseudovirus and incubated for 2 h at 37°C and 5% CO_2_. This mixture was added to the cell culture plates and incubated for 48 h at 37°C and 5% CO_2_. The reading is done on the Spectramax 3 iD (Molecular Devices, LLC, CA, USA) after emptying the plate and filling with firefly luciferase reagent to measure the activity of luciferase which is proportional to the cells infected by the pseudovirus. In this study, this technique was used to assess the neutralizing capacity of AZD7442 against different variants: the wild-type SARS-CoV-2 spike protein (D614G), the alpha (UK B.1.1.7), beta (South Africa B.1.351), gamma (Brazil P.1), delta (Indian B.1.617.2), and omicron subvariants BA.5 and XBB.1.5. The antibody titer was defined as the dilution of serum at which 50% of the infectivity potency is inhibited (IC_50_) using a nonlinear sigmoid model. A sample is considered negative if its dilution titer is below the 20-fold dilution. This technique has already been described in detail elsewhere [27].

#### 2.1.2. Binding Antibodies

Total binding antibodies against the receptor-binding domain (RBD) of the S1 subunit of the SARS-CoV-2 spike protein were measured by the Elecsys anti-SARS-CoV-2 S assay which measured total antibodies (application code *n*°:10230, Roche Diagnostics, Machelen, Belgium) with a positivity cutoff of 0.8 BAU/mL. The analyzer performs an automatic 100-fold dilution for the signal above 250 BAU/mL to extend the measurement range up to 25,000 BAU/mL. In addition, total antibodies against the nucleocapsid (Roche Diagnostics) were measured using the Elecsys anti-SARS-CoV-2 assay. Results above 0.165 cutoff index were considered positive as previously determined [26].

### 2.2. Statistical Analysis

The mean and 95% confidence intervals (95% CI) were used to describe the data. Differences in antibody titers between groups were analyzed using a one-way ANOVA with the Geisser–Greenhouse correction. Correlations were computed using a nonparametric Spearman correlation test. Kinetic models were computed using non-log-transform data and using the following equation: (*a* × *b*)/[(*a*–*b* × basal response) × Exp (−days since vaccination × *c*)] + [*b* × Exp (days since vaccination × *d*)]. In this equation, “*a*” stands for the maximal antibody response, “*b*” for the baseline response, “*c*” for the antibody production rate, and “*d*” for the antibody elimination rate. The half-life time (*T*_1/2_) was obtained from this model, which permitted the calculation of the elimination rate of the antibodies. The area under the curve (AUC) from day 0 to day 90 has been computed. The time required to reach the negativity threshold of the pVNT tests, i.e., a dilution titer below 1/20, was also computed. The significance level was set at a *p* value < 0.05. Analyses were performed using GraphPad Prism 9.0.1 (GraphPad Software, San Diego, CA, USA) and JMP Pro 16.0.0 (JMP®, version 16.0.0 SAS Institute Inc., Cary, NC, USA, 1989-2023).

## 3. Results

### 3.1. Comparison of AZD7442 Patients and the Primo-Vaccination Cohort against the Wild-Type SARS-CoV-2

**Figure 1:**
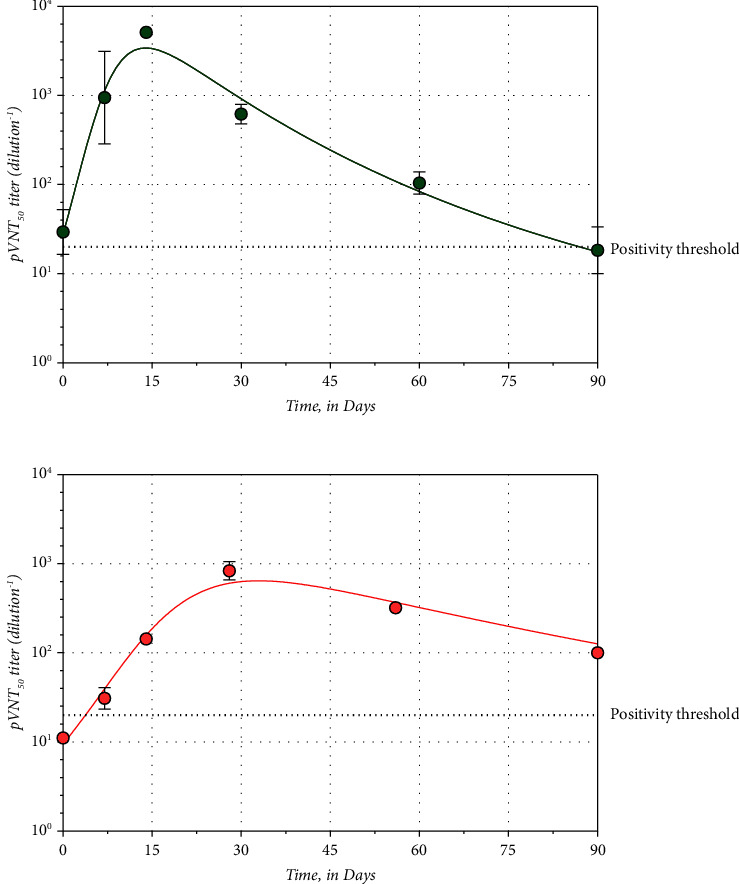
Kinetics models of neutralizing antibodies' response in (a) AZD7442 cases and (b) primo-vaccination BNT162b2 cohort. Means and standard deviation are shown whenever possible for different time points. The dotted line represents the positivity test of the pVNT technique, i.e., an NAb titer of 20.

The maximal NAb titer (in both subjects) was reached after 14 days with AZD7442 (WT NAb titer: 5,120, 95%: not calculable, maximal dilution of the test reached) and after 28 days in the subject with BNT162b2 (WT NAb titer: 2,137 (95% CI: 1,431–3,259) for the primo-vaccinated subjects. The *T*_1/2_ for AZD7442 cases was 8.1 days (95% CI: 5.1–19.5 days) and 11.8 days (95% CI: 7.9–23.7 days) for the primo-vaccination cohort. The time to reach negativity was longer in the vaccination cohort compared to AZD7442 with 108.3 days (95% CI: 66.9–130.7) and 95.4 days (95% CI: 31.0–119.7 days), respectively (Figure 1). The AUC from day 0 to day 90 for the AZD7442 cases was 66,752 pVNT titer dilution^−1^ × day (95% CI: 27,363–164,389) compared to 78,857 pVNT titer dilution^−1^ × day (95% IC: 42,697–118,382) for the vaccination cohort (*p* value > 0.05).

### 3.2. AZD7442 Neutralizing Capacities among Different SARS-CoV-2 Variants

**Figure 2:**
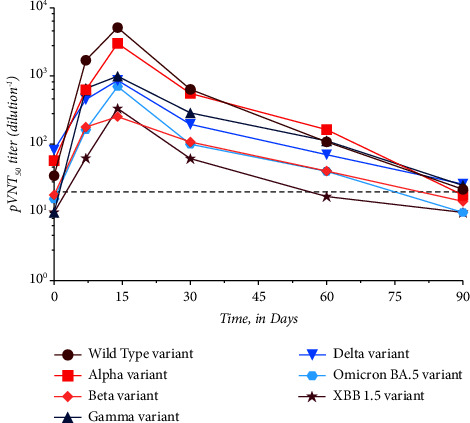
Evolution of the neutralizing antibody (NAb) titers over time following administration of AZD7442 according to the variant of interest. The dotted line represents the positivity threshold of neutralizing antibodies: 1/20.

Maximum titers were all reached 14 days after administration of AZD7442, regardless of the variant. The maximum mean titers differed depending on the variant. For the WT SARS-CoV-2, the maximum mean titer reached the upper limit of the test, i.e., 5,120, while for the alpha variant and the beta variant, maximum mean titers were 3,010 and 253.6, respectively. Regarding the gamma and delta variants, the maximum mean titers were 993.8 and 864.8. Finally, for the omicron BA.5 and XBB.1.5 subvariants, the maximum mean titer was 701.9 and 335.0, respectively (Table 1 and Figure 2).

There was no significant difference between the titers obtained at the different time points with the different variants except after 14 days (*T*_max_). At *T*_max_, there was a significant difference between the NAb titers obtained for the beta, gamma, lambda, omicron BA.5, and XBB 1.5 compared to the WT strain (*p* value < 0.05). Only the alpha variant does not have a significantly different titer compared to the WT strain (*p* value = 0.88) (Table 1). The time to negative differs between variants with the maximum value obtained for the alpha variant (i.e., 101.1 days (95% CI: 30.0–135.4 days)) and the minimum obtained for the beta variant, i.e., 61.2 days (95% CI: 37.8–77.1 days). For the other variants, the WT SARS-CoV-2 had a time to negative of 95.4 days (95% CI: 31.0–119.7 days), the gamma variant 72.9 days (95% CI: 48.7–85.4 days), the delta variant 72.8 days (95% CI: 41–89.2 days), the omicron BA.5 variant 74.1 days (95% CI: 30.7–99.2 days), and the XBB 1.5 variant 65.9 days (95% CI: 29.3–92.1 days).

### 3.3. AZD7442 Total Binding Antibody

**Figure 3:**
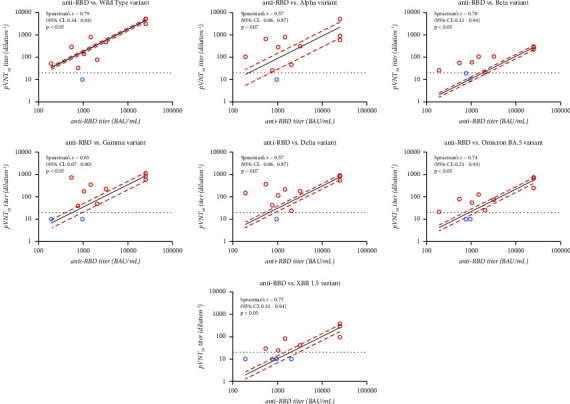
Graphical representation of the correlation between the neutralizing antibody titers obtained in pVNT for the different variants and the total anti-RBD antibody titers obtained with the Elecsys anti-SARS-CoV-2 assay (Roche). Spearman's *r* represents the correlation and its 95% confidence interval (95% CI) is associated with each graph. Dots represented the results of AZD7442 individuals. Red dots are those for which a positive result was obtained for both techniques, while the blue dots represent a negative result in pVNT but positive in total antibodies.

The total binding antibody titer reached the maximum measurable signal (25,000 BAU/mL) after 7 and 14 days after AZD7442 administration for seronegative and seropositive subjects, respectively. However, there was no significant difference in total antibody titers between the two cases. The correlation between anti-RBD total binding antibodies and NAb titer is shown in Figure 3.

Among the different correlations computed, two of them were not significant (i.e., alpha and delta variants, *p*=0.07). The highest correlation was observed for the WT strain (Spearman's *r* = 0.79, 95% CI: 0.34–0.95, *p* < 0.05), and the lowest correlation was observed for the alpha and delta variants (Spearman's *r* = 0.57, 95% CI: −0.06–087, *p*=0.07).

## 4. Discussion

According to our results, AZD7442 provided a higher maximal neutralizing capacity response than the level of NAbs obtained following primary vaccination. The *T*_max_ was also shorter after the injection of mAbs compared to the vaccination (14 vs. 28 days). This rapid and sharp elevation of NAb titers agrees with the data of Levin et al. that reported NAb positivity at 8 days postinjection with the WT strain with a NAb titer of 493.1 (95% CI: 469.3–518.1). However, they reported an increase up to day 28 with a titer of 677.3 (95% CI: 647.1–709.0) [13]. Our results indicate an earlier peak of NAbs at 14 days as well as an earlier decay starting before 28 days. These results are in accordance with those of Loo et al. who reported a similar *T*_max_ at 14 days [30]. Bruel et al. reported positivity in 5 out of 8 patients at three days after injection and in 7 out of 8 patients between 3 and 30 days after administration of AZD7442 [31]. Another major difference between those who received conventional vaccination and those who received mAbs was the elimination kinetics of the NAbs. According to our results, the *T*_1/2_ of the neutralizing capacity induced by AZD7442 was 8.1 days and 11.8 days for the NAbs generated by vaccination, i.e., T1/2 approximately 1.5 times lower. AstraZeneca reported a *T*_1/2_ of 80 days for tixagevimab and 84 days for cilgavimab in the summary of product characteristics (SmPCs) [11]. This may be related to the difference between the measurement of the circulating concentration of AZD7442 and the measurement of the neutralizing capacity induced by the injection of this product, the latter being the technique used in our study. These differences between primary vaccination and administration of AZD7442 raise questions about the prescription of these mAbs. According to the SmPCs, the combination of tixagevimab plus cilgavimab is indicated for both the prophylaxis and treatment of SARS-CoV-2 infections [11]. However, the time to negative reported for the different variants shows a loss of early protection ranging from 101.1 days (days (95% CI: 30.0–135.4 days) for the alpha variant to 65.9 days (95% CI: 29.3–92.1 days) for the XBB 1.5 variant, which raises questions about the product's indication for long-term prophylaxis. These results contrast with those reported in the PROVENT study (conducted before the emergence of omicron and its subvariant), which reported protection for up to 180 days postadministration [13]. These worries about the use of AZD7442 for prophylaxis are shared by the expert panel of the National Institutes of Health responsible for guidelines relating to COVID-19 treatment. In the latest update (March 6, 2023), the expert panel decided on the use of the combination of tixagevimab plus cilgavimab for preexposure prophylaxis of COVID-19 [32]. The current recommended administration schedule for AZD7442 consists of a single 150 mg dose of tixagevimab followed immediately by a single 150 mg dose of cilgavimab. This administration schedule applies to both prophylactic and therapeutic uses. As mentioned by the company, there are no data on the safety and efficacy of repeated doses, but our results suggest that in order to maintain protection against the latest VOIs, several close doses are necessary with a regimen of 1 dose every 2 months [11]. Vaccination and injection of monoclonal antibodies are two different strategies, on the one hand, 150 mg of tixagevimab and 150 mg of cilgavimab could represent more antibodies than the body produces, but on the other hand, the response produced postvaccination is more sustained, demonstrating a continuous production process. This hypothesis is supported by the significant difference between AUCs calculated with a higher AUC for vaccine response (66,752 pVNT titer dilution^−1^ × day (95% CI: 27,363–164,389) compared to 78,857 pVNT titer dilution^−1^ × day (95% IC: 42,697–118,382)).

Among the different correlations computed between NAbs and binding antibodies, there are two of them that are not significant, for the alpha variant and delta variant (*p* value = 0.07). Moreover, there are discrepancies between the two tests for the results obtained at day 90. Indeed, the total antibodies always provide a positive result, while the NAbs are negative (Figure 3). This result description suggests that binding antibodies alone cannot be considered a marker of protection against reinfection. This discrepancy between NAbs and total antibodies has already been reported in the vaccinology literature [18, 33, 34].

At the beginning of the pandemic, CCPs were presented as a solution to the lack of effective therapies. The comparison between CCPs and mAbs is mandatory because CCPs represent an alternative to mAbs administration whereas vaccination is not. Our results show a decrease in the neutralizing capacity of the mAbs tested as a function of the variants analyzed, with the lowest peak in NAbs obtained for the beta variant and the lowest time to negative for the most recent VOI, XBB.1.5. This trend towards a gradual decrease of neutralizing capacity would require the mAbs to be adapted to the circulating VOIs, but this would mean creating new mAbs where the CCPs are, by definition, naturally adapted to the VOIs currently circulating in the population. Franchini et al. observed that CCPs are adapted to the VOIs circulating at the time of plasma collection [35]. However, this also requires frequent collection of CCPs in order to have adequate stocks [36].

## 5. Conclusions

We show a difference in the neutralizing capacity of AZD7442 recipients compared to a vaccinated cohort. This difference can be explained by the mechanism of action of the two approaches. Indeed, mAbs are directly related to their *T*_1/2_, whereas vaccination allows the immune system to generate its own antibodies. Whether with vaccination or mAbs, a modification of the neutralizing capacity according to the VOIs was observed. This observation strengthens the need to assess the effectiveness of these antibodies in patients who have received AZD7442 over time. Our results reinforce the need of reviewing the use of AZD7442 in relation to VOIs and potentially adapting its administration schedule. Based on these and depending on the variant, it could be of interest to consider a bimonthly administration of AZD7442, especially considering the XBB1.5 variant. AZD7442 could be indicated for short-term prophylaxis in frail patients who may be acutely exposed to SARS-CoV-2. Nevertheless, further investigations are necessary to confirm these results, and continuous surveillance of VOIs is needed.

## Figures and Tables

**Table 1:** Mean neutralizing antibody titers after AZD7442 administration were obtained for each time point with the different SARS-CoV-2 variants tested.

SARS-CoV-2 variant	Wild-type pVNT mean titer (titer dilution^−1^)	Alpha pVNT mean titer (titer dilution^−1^)	Beta pVNT mean titer (titer dilution^−1^)	Gamma pVNT mean titer (titer dilution^−1^)	Delta pVNT mean titer (titer dilution^−1^)	Omicron BA.4/5 pVNT mean titer (titer dilution^−1^)	XBB 1.5 pVNT mean titer (titer dilution^−1^)
Days since AZD7442 administration							
0	34.47	57.41	18.09	10.00	82.80	15.75	10.00
7	1704.00	627.20	178.50	656.60	453.20	163.20	62.29
14	5120.00	3010.00	253.60	993.80	864.80	701.90	335.00
28	638.10	555.00	107.80	287.70	195.80	100.00	61.58
56	108.50	163.70	40.52	112.10	70.36	40.00	17.11
90	21.82	17.97	14.54	24.82	25.99	10.00	10.00

## Data Availability

The data used to support the findings of this study have not been made available because of other investigations on the project.
